# Grasping tiny objects

**DOI:** 10.1007/s00426-024-01947-8

**Published:** 2024-03-30

**Authors:** Martin Giesel, Federico De Filippi, Constanze Hesse

**Affiliations:** 1 School of Psychology, University of Aberdeen, William Guild Building, Aberdeen, AB24 3FX UK; 2 School of Psychology and Neuroscience, University of St Andrews, St Mary’s Quad, South Street, St Andrews, KY16 9JP UK

## Abstract

In grasping studies, maximum grip aperture (MGA) is commonly used as an indicator of the object size representation within the visuomotor system. However, a number of additional factors, such as movement safety, comfort, and efficiency, might affect the scaling of MGA with object size and potentially mask perceptual effects on actions. While unimanual grasping has been investigated for a wide range of object sizes, so far very small objects (<5 mm) have not been included. Investigating grasping of these tiny objects is particularly interesting because it allows us to evaluate the three most prominent explanatory accounts of grasping (the perception-action model, the digits-in-space hypothesis, and the biomechanical account) by comparing the predictions that they make for these small objects. In the first experiment, participants ($N=26$) grasped and manually estimated the height of square cuboids with heights from 0.5 to 5 mm. In the second experiment, a different sample of participants ($N=24$) performed the same tasks with square cuboids with heights from 5 to 20 mm. We determined MGAs, manual estimation apertures (MEA), and the corresponding just-noticeable differences (JND). In both experiments, MEAs scaled with object height and adhered to Weber’s law. MGAs for grasping scaled with object height in the second experiment but not consistently in the first experiment. JNDs for grasping never scaled with object height. We argue that the digits-in-space hypothesis provides the most plausible account of the data. Furthermore, the findings highlight that the reliability of MGA as an indicator of object size is strongly task-dependent.

**Supplementary Information:**

The online version contains supplementary material available at 10.1007/s00426-024-01947-8.

## Introduction

When reaching towards an object that we want to grasp, e.g., with thumb and index finger (i.e., precision grip), the digits are opening towards an object-dependent maximum distance between them (maximum grip aperture, MGA). This maximum is usually reached after 75–80% of the movement time (Jeannerod, 1984; Smeets and Brenner, 1999). After the maximum has been reached, the digits close again to finally make contact with the object at the contact points (Jeannerod, 1981). In general, the MGA exceeds the boundaries of the object because it also contains a safety margin to allow the digits to safely manoeuvre around the edges of the object to reach the contact points without colliding with other parts of the object (Jeannerod, 1981, 1984). When in the following we talk about grasping, we always refer to grasping movements that generally follow this pattern.

Broadly, there are two different views about which cues are used to program the MGA. Jeannerod (1981) found that the MGA varies linearly with the size of objects. In most subsequent studies, the MGA has been regarded as representing the visual size estimate of the grasp target. While the MGA is not an accurate measure of object size, because it also contains the safety margin, it has been shown to accurately reflect size differences between objects (Hesse & Franz, 2009; Jeannerod, 1981; Marteniuk et al., 1990; Smeets & Brenner, 1999). Smeets and Brenner (1999) on the other hand, proposed that grasping is guided by the positions of the contact points on the grasp targets. They suggested that the digits independently point to and move towards those contact points (digits-in-space hypothesis; for a review see Smeets et al., 2019).

Independent of these two broad views about which cues are used to program the MGA, there are additional factors that might modulate the MGA or the choice of cues used to program the MGA. Here, we focus on a group of factors referred to as biomechanical constraints. The way we reach towards an object, and how during the reach the hand is shaped to grasp the object, is not only determined by the intrinsic (e.g., size, shape, weight) and extrinsic (e.g., orientation, position) properties of an object but also by anatomical features and physiological demands (Schenk et al., 2017; Utz et al., 2015). We distinguish between hard and soft biomechanical constraints: Hard biomechanical constraints refer to anatomical limits such as the maximal hand span. Soft biomechanical limits refer to a tendency to make efficient, comfortable and safe movements if violations of these tendencies are not specifically required by the task demands. In general, with increasing object size, increasing effort is required to reach the MGA. This might conflict with the soft biomechanical constraint that we strive to perform comfortable, safe, and efficient movements (Rosenbaum et al., 2001; Schenk et al., 2017). If an object’s size is close to the hand span or exceeds it, the object cannot be safely grasped anymore, i.e., the safety margin is reduced or non-existent, and possibly a different type of grasp or grasping strategy has to be employed.

Furthermore, the variability of MGAs in repeated grasps of the same object has been used to study if grasping follows a fundamental psychophysical principle, i.e., Weber’s law. Weber’s law postulates that JNDs linearly increase with stimulus magnitude. The variability of MGAs is usually determined by computing the standard deviation of the MGAs and is used to estimate the just-noticeable differences (JNDs) for the visual cue assumed to be used to program the grasping movement, e.g., size or position (for a different view see Bhatia et al., 2022).

In a seminal paper, Ganel et al. (2008) asked participants to perform two tasks: a perceptual and a visuomotor task. In the perceptual task, they were asked to provide repeated manual estimates of the size of objects, i.e., participants adjusted their index finger and thumb so that the distance between them represented the perceived size of an object. In the visuomotor task, participants were asked to repeatedly grasp the same objects. JNDs were computed for the manual estimates and MGAs. While the JNDs for manual estimation followed Weber’s law, i.e., JNDs linearly increased with object size, the JNDs for grasping remained constant and did not follow Weber’s law. Subsequently, a range of studies confirmed these findings (e.g., Ganel et al., 2014; Hadad et al., 2012; Holmes et al., 2013; Ozana & Ganel, 2019). Assuming that visual size was the cue used in both the manual estimation and grasping tasks, Ganel et al. (2008) interpreted this finding as evidence for a dissociation between perception and action as hypothesised by the perception-action model (PAM; Milner & Goodale 1995, 2006). According to the PAM, the visual input to manual estimation and grasping is processed in two different (largely) independent pathways, the ventral and the dorsal path. While visual information in the ventral path is affected by psychophysical principles, e.g., by Weber’s law, visual information in the dorsal path is not because visual information for grasping has to be precise and metrically accurate.

However, Smeets and Brenner (2008) proposed an alternative explanation for the data of Ganel et al. (2008). They argued that if, as proposed by the digits-in-space hypothesis (Smeets and Brenner, 1999), position and not size is the relevant cue for the programming of the MGA, then one would not expect adherence to Weber’s law because Weber’s law requires a property that has a magnitude (i.e., a property that starts at zero and is non-negative). While this is the case for size, it is not for position data. Finding Weber’s law in the manual estimation task in which participants were explicitly asked to make a size judgement, and not finding it in the grasping task, is consistent with the predictions of the digits-in-space hypothesis.

Another explanation for the apparent absence of Weber’s law in grasping is provided by the biomechanical account (Utz et al., 2015). It does not necessarily question the use of size information in guiding grasping movements but suggests that hard and soft biomechanical constraints modulate the grasping response in a way that might overshadow the effect of Weber’s law. As with increasing object size the hand opening approaches its hard limit, the variability of the aperture is reduced. This reduction in variability can either compensate the increase in variability with size due to Weber’s law resulting in an approximately constant variability (e.g., Ganel et al., 2008) or even outweigh it resulting in a decrease in variability with increasing object size (e.g., Uta et al., 2015). The reason that the same pattern is not observed for the JNDs in manual estimation tasks can be explained by the different task demands. In manual estimation, participants are explicitly asked to make accurate judgements of size. This demand might overrule the tendency to make comfortable and efficient movements. Moreover, since manual estimation does not require a safety margin, a larger range of objects can be comfortably estimated than can be grasped. Support for the biomechanical account is provided by Bruno et al. (2016) and Uccelli et al. (2021) who found that grasping followed Weber’s law for object sizes from 5 to 20 mm. However, for objects in the range from 20 to 120 mm, they found no adherence to Weber’s law, i.e., they found decreasing variability with increasing size. This is also the range in which Ganel et al. (2008) and Utz et al. (2015) reported an absence of Weber’s law in grasping.

The sizes of objects used in most unimanual grasping studies investigating MGAs and JNDs were approximately in the range from 5 to 100 mm (Smeets & Brenner, 2008). Here, we set out to investigate—for the first time—grasping and manual estimation for very small objects with heights ranging from 0.5 to 5 mm (Experiment A). In a different experiment (Experiment B), we tested objects with heights ranging from 5 to 20 mm to replicate the findings by Bruno et al. (2016) and Uccelli et al. (2021) who found that both grasping and manual estimation adhered to Weber’s law for object sizes in this range. Importantly, in all experiments, we presented objects in such a way that the contact points for both the index finger and thumb were visible during movement planning (Volcic & Domini, 2014).

Investigating grasping for very small objects is interesting with respect to the different explanatory accounts of grasping for three main reasons:

- **Ecological validity.** In most grasping studies, participants grasp objects using the precision grip (i.e., grasping with thumb and index finger). In everyday situations, we use this type of grasp mostly only for very small objects (e.g., picking up a hair or pulling-out a credit card from an ATM). The combination of precision grip and small objects therefore has a high ecological validity and relevance.
- **Visual resolution for size and position differences.** This is relevant for evaluating both the predictions of the digits-in-space hypothesis and the predictions of the PAM. In the context of the digits-in-space hypothesis, the resolution for positions is assumed to be lower than the resolution for size (Smeets & Brenner, 2008; Smeets et al., 2020). Within the context of the PAM, Ganel et al. (2012) reported that grasping has a higher resolution for size increments than perception. This supports the assumption that the visual information in the action-related dorsal path is more accurate and precise than the visual information in the perception-related ventral path (but see Göhringer et al., 2019 for a critical methodological re-evaluation of these findings).
- **Biomechanical constraints.** Analogous to the soft and hard biomechanical constraints for large objects, there could also be biomechanical constraints for very small objects. Obviously, the smaller the objects, the closer index finger and thumb come to each other. They represent hard limits for each other that could, in principle, have the same variability-reducing effect as the hard limit of the maximal span between thumb and index finger. Moreover, in the same way as more effort is required to open the fingers as the size of an object approaches the maximal hand opening, pressing the digits together requires more effort the closer the fingers get. Holding thumb and index finger so that there is only a very small opening between them is quite effortful.

Based on these points, we can now derive the predictions that the three explanatory accounts of grasping make for MGAs and JNDs in the case of grasping very small objects.

### Predictions for MGAs and JNDs in the grasping task

**Fig. 1:**
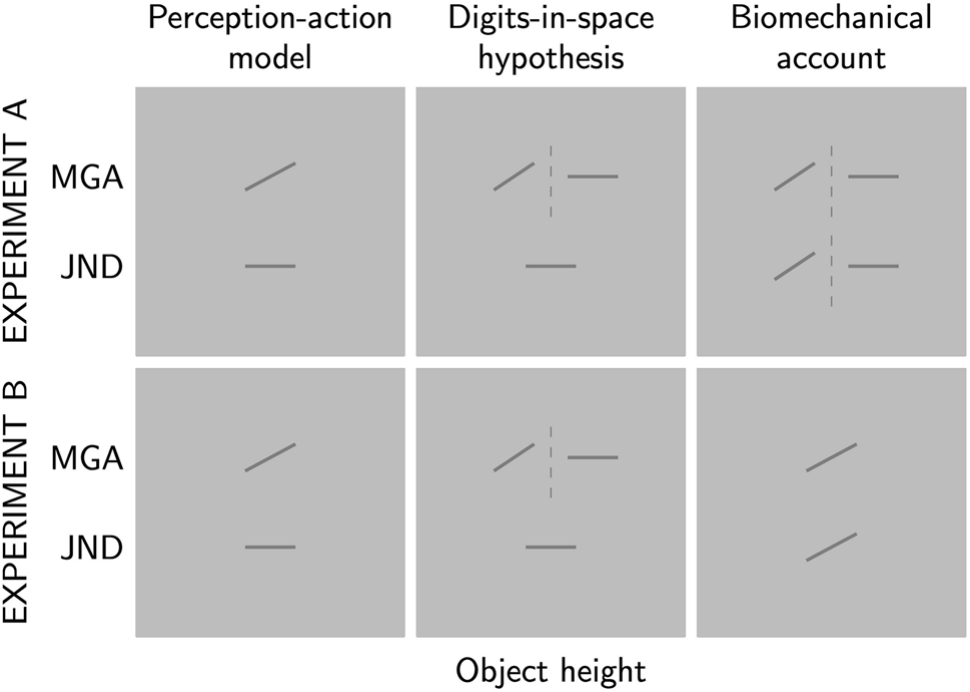
Predictions of three explanatory accounts of grasping—perception-action model (1st column), digits-in-space hypothesis (2nd column), biomechanical account (3rd column)—for the slopes of MGAs and JNDs for grasping for Experiments A (top row) and B (bottom row). In Experiment A, object heights ranged from 0.5 to 5 mm and in Experiment B from 5 to 20 mm. Lines indicate whether MGAs/JNDs linearly increase with object height (lines with slope > 0) or not (lines with slope = 0). See text for details

Figure 1 shows the predictions that the three different explanatory accounts of grasping (i.e., perception-action model, digits-in-space hypothesis, and biomechanical account) make for the MGAs and JNDs in the grasping task of our two experiments. These accounts are not necessarily mutually exclusive. Particularly, biomechanical constraints could affect grasping independent of whether size or position is the relevant cue. Note, that while the digits-in-space hypothesis and the biomechanical account specifically address grasping behaviour, the PAM is a broader model of action and perception that is based on a wide array of experimental and neuropsychological data. When in the following we refer to the PAM, we only refer to it in so far as it was used by Ganel et al. (2008) and others to explain grasping and manual estimation data based on the different properties of processing of visual information in the dorsal and ventral pathways. We are not attempting to evaluate the validity of the PAM in general.

In Experiment A, we determined MGAs, maximal estimation apertures (MEAs), and JNDs for grasping and manual estimation for objects that varied in height in eight steps from 0.5 to 5 mm. In Experiment B, we did the same for objects that varied in height in six steps from 5 to 20 mm. The lines in Fig. 1 indicate whether an account predicts for the two experiments if MGAs and JNDs increase linearly with object height (lines with slope > 0) or if they do not linearly increase with object height (lines with slope = 0), i.e., including MGAs or JNDs decreasing with increasing object height. Note that we only show predictions for the MGAs and JNDs in the grasping task because the three accounts make the same predictions for the MEAs and JNDs in the manual estimation task in both experiments: MEAs and JNDs increase linearly with object height as long as the objects are perceptually discriminable.

The PAM assumes that size is the relevant cue both for manual estimation and grasping. As described above, the visual information in the ventral pathway is allocentric and holistic, i.e., it preserves relative aspects of object dimensions at the cost of absolute metrics. In contrast, the information in the dorsal pathway is processed analytically, i.e., it retains metrically accurate data of the action-relevant object dimensions for objects in the peri-personal space (Ganel & Goodale, 2003, 2014). In our view, this implies (even if this has so far not been explicitly stated by the PAM) that when it comes to the metrically accurate perception of a grasp target (within the workspace), the two systems either produce similarly accurate information, i.e., if the ventral information has not been altered by the holistic processing, or the information in the ventral pathway is less metrically accurate than the information in the dorsal pathway. To our knowledge there is within the PAM no reason why visual information in the dorsal pathway should be less metrically accurate (for objects within the workspace) than visual information in the ventral pathway (but see Discussion). Accordingly, we predict that if the objects can be perceptually discriminated, i.e., if the MEAs linearly increase with height in the manual estimation task, then MGAs in grasping should also increase with height since the resolution for size information provided by the dorsal path should at least be as high as, if not higher than, the resolution provided by the ventral path. Furthermore, the PAM predicts that JNDs for grasping do not increase with object height because the visual information in the dorsal path is not affected by psychophysical principles like Weber’s law.

Since the digits-in-space hypothesis assumes that the relevant cue for grasping is position, and Weber’s law is not expected to apply to position data, JNDs should neither increase with height in Experiment A nor in Experiment B. For the MGAs in Experiments A and B, the predictions depend on whether positions can still be discriminated for the objects. Since the digits-in-space hypothesis assumes that different cues are used in manual estimation and grasping, MEAs in the manual estimation task do not allow making predictions about MGAs in the grasping task. If positions can be discriminated, MGAs should increase with object height, otherwise they should remain constant.

The predictions of the biomechanical account for Experiment B are based on the findings by Bruno et al. (2016) and Uccelli et al. (2021). They found that MGAs and JNDs increased linearly with object size in the range from 5 to 20 mm indicating that in this range the effects of biomechanical constraints are not strong enough to overshadow the effect of Weber’s law. Based on this, we can predict that the same holds true for the smaller objects in Experiment A, i.e, both MGAs and JNDs for grasping should linearly increase with object height. In addition to this prediction, we propose an alternative prediction by taking into account biomechanical constraints that could exist for very small objects. For these very small objects, the combination of height estimate and appropriate safety margin could potentially require programming an MGA considerably narrower than the natural opening between index finger and thumb. The effort to hold such a position might be avoided by not scaling the MGA with very small object heights. If MGAs do not scale with object height, JNDs are also not expected to increase with object height. We found that the results of both experiments are congruent with the predictions of the digits-in-space hypothesis which, in contrast to the two other accounts, can explain the findings without additional assumptions.

## Methods

No part of the study procedures and analyses was pre-registered prior to the research being conducted. We reported how we determined our sample size, all data exclusions, all inclusion/exclusion criteria, whether inclusion/exclusion criteria were established prior to data analysis, all manipulations, and all measures in the study. Experiments A and B only differed in the types of stimuli that were used. All other aspects of the experiments were identical. Different participants participated in the two experiments.

### Participants

In Experiment A, we collected data from 28 right-handed participants (21 female, age range = 18–38 years, mean (SD) age = 22.3 (4.41)). In Experiment B, we collected data from 24 right-handed participants (15 female, age range = 18–39 years, mean (SD) age = 25.5 (5.47)). Participants in Experiment B had not participated in Experiment A. The experimental procedures were in accordance with the Code of Ethics and Conduct and the Code of Human Research Ethics by the British Psychological Society (BPS) and were approved by the School of Psychology Research Ethics Committee of the University of Aberdeen (Ethics code: PEC/4928/2022/2). The participants were naive as to the purpose of the experiment. They had normal or corrected-to-normal vision. All participants gave written informed consent. They were compensated with £10 for their time. The sample sizes of the two experiments were chosen to match or exceed the sample sizes used in the majority of related studies on grasping.

### Setup

**Fig. 2:**
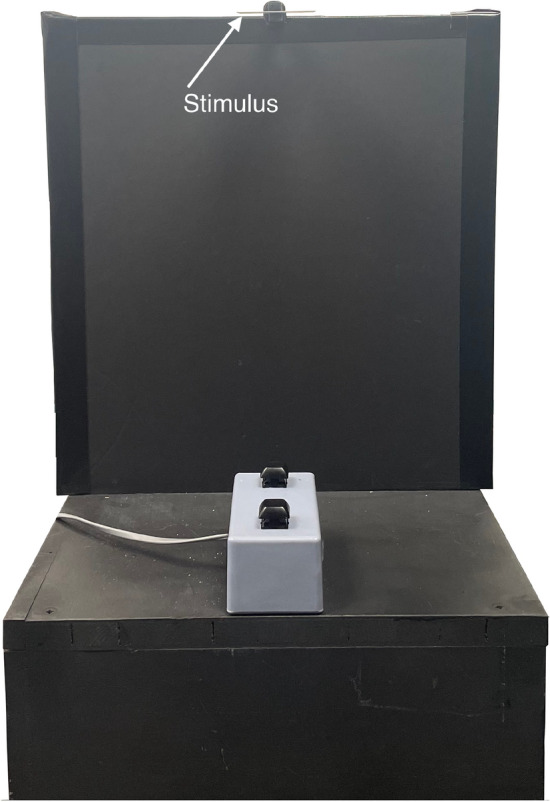
Setup with example stimulus. At the beginning of each trial, the participants’ index finger and thumb rested on the front button of the button box. After an auditory signal, they reached up to grasp the stimulus held in place by a clothes-peg. They placed the stimulus on the table and then placed their finger and thumb back on the front button

Figure 2 shows the setup that was used for all experiments. Participants sat on a height-adjustable chair in front of a wooden table (72 cm high). A wooden platform (11.5 cm high, 30.5 cm wide) was placed on the table. On top of the platform, a two button-box (5.5 cm high) connected to the PC through the parallel port was placed centrally. The front button was used as the start and end position of the movement. A wooden box (35 cm high, 32 cm wide) was placed on top of the first box. At the top of this box, the stimuli were presented, held in place by a wooden clothes-peg painted black. Participants adjusted the chair in such a way that the vertical centre of the stimuli was approximately at eye-level so that both the top and bottom sides of the stimuli were in the participants’ line-of-sight. Stimuli were placed with the small side (height dimension) facing the participants. The direct distance between the fingers’ start position and the front of the stimulus was approximately 30 cm.

A TrakStar electromagnetic motion-tracker (Ascension Technology Corporation, NDI) with a sampling rate of 240 Hz was used to track the movements of the participants’ index finger and thumb. Markers were placed on the fingernail of the right index finger and thumb. They were attached with white-tac and medical tape without covering the finger pads. Velcro-strips were used to secure the wires.

Participants’ vision was occluded between trials and after movement onset using liquid crystal shutter goggles (PLATO Translucent Technologies, Toronto, Ontario, Milgram, 1987). The experiment was programmed in Matlab (Mathworks, Natick, MA, USA).

### Stimuli

**Fig. 3:**
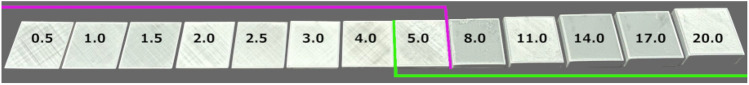
Stimuli used in Experiment A (0.5–5 mm, magenta outline) and Experiment B (5–20 mm, green outline). The width and depth of all stimuli was 5 $\times$ 5 cm

Figure 3 shows the stimuli used in Experiments A and B. In both experiments the stimuli were 3D printed blocks (5 cm wide and deep) made from grey polylactic acid (PLA) filament. The blocks only differed in height. In Experiment A, blocks with eight different heights (0.5, 1.0, 1.5, 2.0. 2.5, 3.0, 4.0, 5.0 mm) were used (magenta outline in Fig. 3). In Experiment B, blocks with six different heights (5.0, 8.0, 11.0, 14.0, 17.0, 20.0 mm) were used (green outline in Fig. 3). Note that one height (5.0 mm) was used in both experiments. To fit into the opening of the clothes-peg that held the stimuli in place, for the blocks with heights of 8.0, 11.0, 14.0, 17.0, and 20.0 mm a groove was cut into the back of those blocks. With a viewing distance of approximately 45 cm and a Weber fraction of k=0.06 for visual size perception (Smeets & Brenner, 2008), all object heights were assumed to be perceptually distinct.

### Procedure

In both experiments, participants performed two different types of tasks: a grasping task and a manual height estimation task. The order in which they completed these tasks was counterbalanced. Half of the participants started with the grasping and half started with the manual estimation task. Within each task, stimuli were presented in pseudo-randomised order. To avoid that hand aperture adjustments are influenced by visual feedback during movement execution, grasping and manual estimation were performed open-loop, i.e., there was no visual feedback after movement onset (see Bruno et al., 2016).

#### Grasping task

In the grasping task, participants were asked to grasp the different stimuli with thumb and index finger (precision grip). At the beginning of each trial, thumb and index finger pressed down the front button of the button box, and the shutter glasses were closed. The experimenter started a trial by pressing a button. After one second, the shutter glasses opened for a one second preview period. An auditory signal indicated to the participants when they should start reaching towards the stimulus. As soon as the button was released, the shutter glasses closed. The participants reached towards the stimulus, grasped it with index finger and thumb, pulled it out of the clothes-peg and placed it on the table. Then they returned to the start position on the button box. Between trials, the experimenter exchanged the stimuli. Each stimulus was presented 10 times (Experiment A: 8 stimuli $\times$ 10 repetitions = 80 trials; Experiment B: 6 stimuli $\times$ 10 repetitions = 60 trials). Participants performed 8/6 practise trials before data collection started.

#### Manual height estimation task

In the manual height estimation task, participants were asked to indicate the height of the stimuli with their index finger and thumb without touching the stimulus. At the beginning of each trial, thumb and index finger pressed down the front button of the button box, and the shutter glasses were closed. The experimenter started a trial by button-press. After one second, the shutter glasses opened for a one second preview period. An auditory signal indicated to the participants when they should start the estimation. When they lifted index finger and thumb from the button, the shutter glasses closed. They then adjusted the distance between index finger and thumb so that it represented the height of the stimulus. Once they were satisfied with the adjustment, they were asked to hold that position and verbally indicated to the experimenter that they had finished the adjustment. The experimenter then recorded the index finger and thumb positions by button-press. After that the glasses opened, and the participants were asked to briefly touch the stimulus without removing it. Then they placed their fingers back on the start button, and the shutter glasses closed. Between trials, the experimenter exchanged the stimuli. Each stimulus was presented 10 times (Experiment A: 8 stimuli $\times$ 10 repetitions = 80 trials; Experiment B: 6 stimuli $\times$ 10 repetitions = 60 trials). Participants performed 8/6 practise trials before data collection started.

### Data analysis

In all experiments, the 3D trajectories for the index finger and thumb were filtered offline using a second-order Butterworth-filter with a low-pass cut-off frequency of 15Hz. Resultant velocity was calculated from the filtered 3D position data of the markers. Movement onset was defined as the time point at which the button was released and movement end was determined, using a velocity criterion, as the first time point after movement onset where either the thumb or index finger velocity was below 0.075 mm/ms.

Exclusion criteria were selected in line with our previous grasping studies (e.g., Giesel et al., 2020). In the grasping tasks, trials were excluded when the movements started before the auditory start signal, reaction time was too short (<100 ms), or the movement time was too long (>1500 ms). In the manual estimation tasks, trials were excluded when the movements started before the auditory start signal, the reaction time was too short (<100 ms), or when participants took more than 5 s to provide an estimate. If during the experiment trials met these exclusion criteria, the trials were classified as an error and repeated later in the experiment at a random position. Additionally, in Experiment A three estimation and two grasping trials and in Experiment B two grasping trials were excluded offline from the data analysis. In Experiment A, two participants were excluded from the data analysis because they had not followed the instructions and not closed index finger and thumb at the beginning of the trials.

The MGA was computed as the maximum 3D Euclidean distance between the thumb and index finger markers reached during the grasping movement. For manual estimation, the 3D Euclidean distance was computed for the MEA, i.e., the 3D Euclidean distance between index finger and thumb markers at the time when the estimate was recorded. For both experiments, we computed linear regressions using a least-squares criterion for MGAs, MEAs and the corresponding JNDs separately for each participant. For each object height, each participant performed 10 grasping and 10 estimation trials (see the grey circles in Figures S1–S4 in the Supplemental material). Regression lines were fitted separately for each participant to these MGAs/MEAs (see the red lines in Figures S1–S4). Following the literature (Ganel et al., 2008), JNDs were computed as the standard deviation of the 10 MGAs and MEAs each participant produced for each stimulus height. Then regression lines were fitted separately for each participant to the JND values.

The data was analysed using both frequentist statistical tests and their Bayesian equivalents. The Bayesian analysis follows the recommendations of van Doorn et al. (2021). To compare if the slopes for MGAs, MEAs, and JNDs differed between the two tasks (grasping vs estimation), two-sided paired-samples *t*-tests were used. Since the predictions for the different models (see Fig. 1) were directional (lines with slope = 0 or lines with slope > 0 in Fig. 1), these predictions were tested using one-sided one-sample *t*-tests. Note that using two-sided one-sample *t*-tests would not result in a change of the conclusions. All Bayesian *t*-tests (JASP Team, 2023; Morey & Rouder, 2015; Rouder et al., 2009) were performed with the default Cauchy prior width of 0.707. For the Bayesian *t*-tests, we report here only the Bayes factor, but for each test the complete analysis including plots of the prior and posterior distributions and Bayes factor robustness checks are provided as JASP files on OSF. The Bayes factor provides a measure of the strength of evidence for one hypothesis relative to another. For two-sided *t*-tests, BF_10_ indicates the Bayes factor in support of the alternative hypothesis compared to the null hypothesis, whereas BF_01_ is the Bayes factor in support of the null hypothesis over the alternative hypothesis, with BF_10_=1/BF_01_. For one-sided *t*-tests, BF_+0_ indicates the Bayes factor in support of the alternative hypothesis over the null hypothesis. The Bayes factor is a continuous value ranging from 0 to $\infty$, with a Bayes factor of 1 indicating that both hypotheses have equal support in the data. A frequently used classification of Bayes factors (Jeffreys, 1961) considers Bayes factors between 1 and 3 as weak/anecdotal evidence, between 3 and 10 as moderate evidence, and Bayes factors larger than 10 as strong evidence. Statistical analysis was performed in Matlab and JASP (JASP Team, 2023). JASP files for the data analysis performed here are available from the OSF (https://osf.io/jv7as/).

## Results

### Experiment A

We first present the analysis for MGAs and MEAs and then for JNDs computed as the standard deviation of the MGAs/MEAs.

#### MGAs & MEAs

**Fig. 4:**
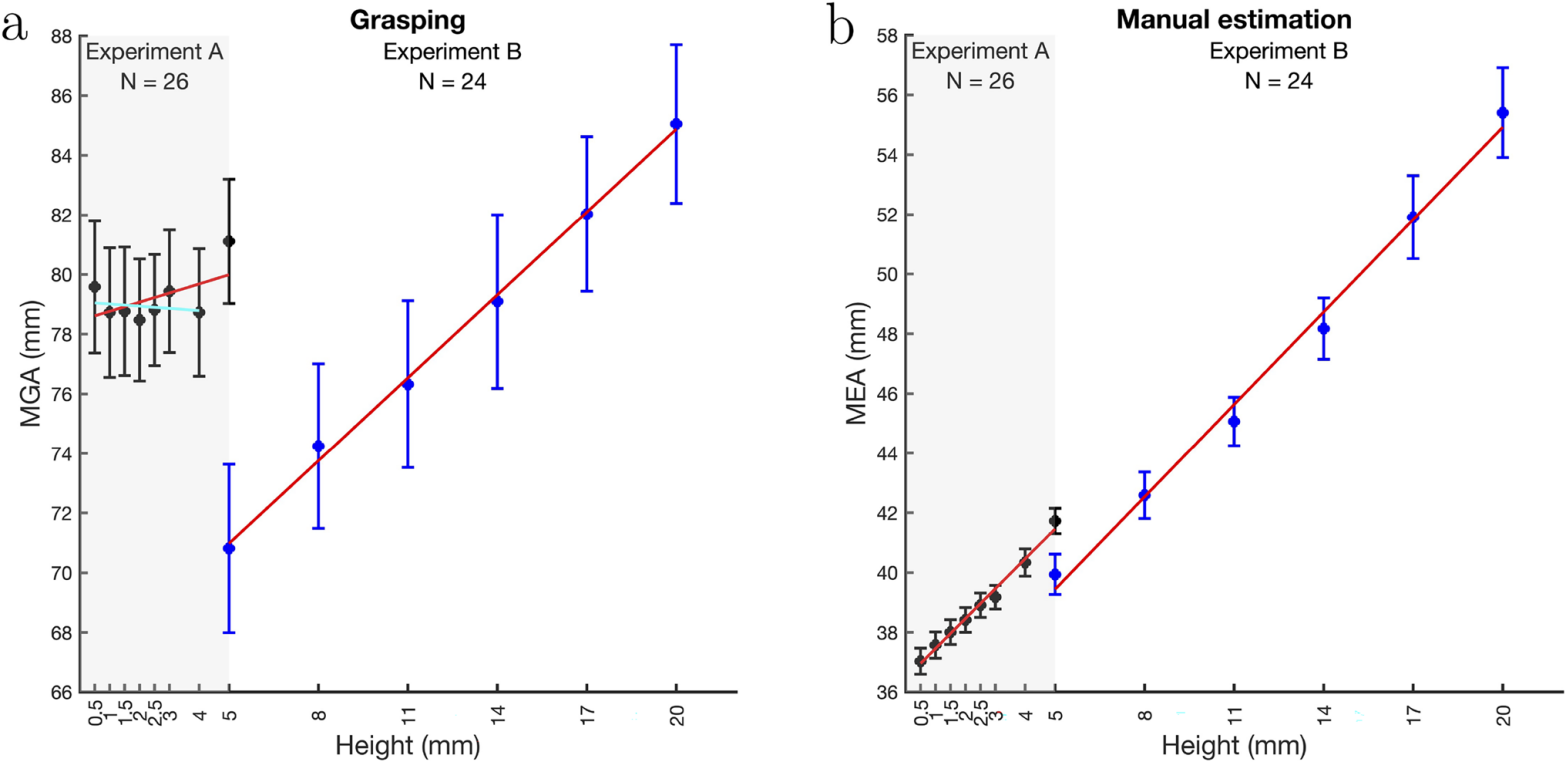
MGAs (**a**) and MEAs (**b**) for Experiment A (black data points) and Experiment B (blue data points). Error bars show ±1 SEM (between-subjects). Red lines are regression lines based on the averaged slopes and intercepts resulting from fits to individual participants’ MGAs/MEAs (also see Figures S1–S4 in the Supplemental material). The cyan line is the regression line fitted to the MGAs for object heights from 0.5 to 4 mm, i.e., excluding the highest object (5 mm). Note that MGAs/MEAs include the height of index finger and thumb and of the two markers sitting on top of the digits

The black data points in Fig. 4 show the MGAs for grasping (Fig. 4a) and MEAs for manual estimation (Fig. 4b) for Experiment A averaged over participants. Table 1 shows the slopes for MGAs and MEAs (M ± 1 SEM) averaged over participants. The slope for manual estimation was steeper than the slope for grasping.

**Table 1:** Slopes (M ± 1 SEM) for grasping and manual estimation for Experiments A and B

		MGA/MEA	JND_STD_
Experiment A	Grasping	0.31 ± 0.15	− 0.17 ± 0.08
	(0.5–4 mm)	− 0.07 ± 0.16	
	Estimation	1.00 ± 0.07	0.16 ± 0.02
	(0.5–4 mm)	0.91 ± 0.07	
Experiment B	Grasping	0.93 ± 0.07	− 0.01 ± 0.03
	Estimation	1.03 ± 0.08	0.16 ± 0.04

There was a significant difference between the slopes for grasping and manual estimation (t(25) = $-$4.824, p < .001, d = $-$0.946, BF_10_ = 430.856). A one-sided one-sample *t*-test showed that the slope for grasping was significantly larger than zero (t(25) = 2.068, p = .025, d = 0.406, BF_+0_ = 2.485). The Bayes factor indicates that the support for the hypothesis that the slope is larger than zero is anecdotal to moderate. Figure 4a suggests that this non-zero slope might be largely due to the value for the highest stimulus (5 mm). Note that 5 mm is the smallest stimulus size used in comparable previous studies which consistently observed scaling to object size for stimuli larger than 5 mm (Bruno et al., 2016). Somewhere between object heights of 4 and 5 mm there might be a transition point where participants start scaling MGAs with object height. To test this (unplanned post-hoc comparison), we also determined regression line fits to only the stimuli with heights from 0.5 to 4 mm, i.e., excluding the 5 mm stimulus (see cyan lines in Fig. 4a and Figure S1 in the Supplemental material). In this case, the slope averaged over participants is M=$-$0.07 ± 0.16. The one-sided one-sample *t*-test testing whether the slope was larger than zero was not significant (t(25) = $-$0.459, p = .675, d = $-$0.090, BF_+0_ = 0.151). This might indicate that between stimulus heights of 4 and 5 mm, the MGA starts scaling with object size but is constant for smaller objects. The slope for manual estimation was significantly larger than zero (t(25) = 15.231, p < .001, d = 2.987, BF_+0_ = 3.5$\times$10^+11^). Removing the highest stimulus from the regression line fits changes the mean slope to M = 0.91 ± 0.07 which is still significantly different from zero (t(25) = $-$13.769, p < .001, d = 2.700, BF_+0_ = 4.1$\times$10^+10^). Although not part of our model predictions, but of general interest, we also tested if the slope for manual estimation was one. The two-sided Bayesian *t*-test showed moderate support for the null hypothesis that the slope was one (BF_01_ = 4.825). The equivalent two-sided frequentist *t*-test was not significant (t(25) = $-$0.019, p = .985, d = $-$0.004).

The slope close to one for the MEAs shows that participants accurately perceived the height differences between the different objects. The differences between the slopes of MGAs and MEAs suggest that the resolution of the perceptual system is higher than that of the visuomotor system and/or that there are other reasons why grasping may not scale MGAs to very small object sizes.

#### JNDs

**Fig. 5:**
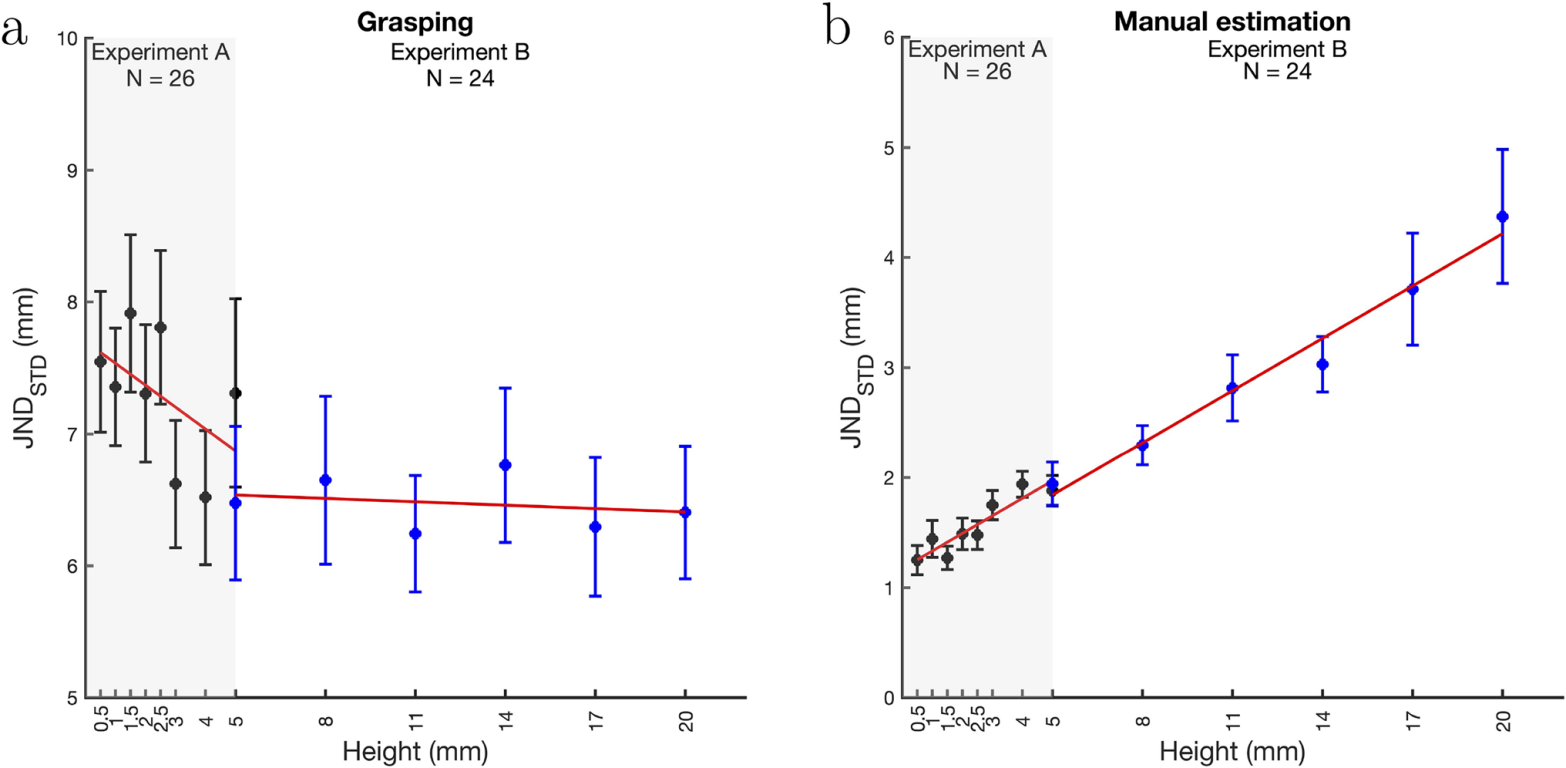
JNDs for grasping (**a**) and manual estimation (**b**) for Experiment A (black data points) and Experiment B (blue data points). JNDs were computed as the mean of the standard deviations of individual participants’ MGAs/MEAs. Error bars show ±1 SEM (between-subjects). Red lines are regression lines based on the averaged regression parameters resulting from fits to individual participants’ JNDs

The black data points in Fig. 5 show averaged JNDs for Experiment A computed as the standard deviation of the individual participants’ MGAs (Fig. 5a) and MEAs (Fig. 5b).

JNDs differed between manual estimation and grasping with JNDs for manual estimation being much smaller than the JNDs for grasping. Notably, the JNDs for grasping also show much higher variability between participants than the JNDs for manual estimation. Table 1 shows the slopes for JNDs averaged over participants. There was a significant difference between the slopes for grasping and manual estimation (t(25) = $-$4.001, p < .001, d = $-$0.785, BF_10_ = 64.043). As indicated by the Bayes factor, the slope for grasping was not larger than zero (t(25) = $-$2.052, p = .975, d = $-$0.402, BF_+0_ = 0.076). The slope for manual estimation increased with object size and was significantly larger than zero (t(25) = 6.601, p < .001, d = 1.295, BF_+0_ = 53365).

Together with the positive slope for the MEAs, this indicates that manual estimation for very small objects follows Weber’s law. Since for grasping, the MGAs only scaled weakly with object height, and not at all when the highest stimulus was removed, it is unsurprising that we found no adherence to Weber’s law. Both the results for MGAs and JNDs for grasping seem consistent with the differences in object heights being below the resolution of the visuomotor system.

### Experiment B

As for Experiment A, we first present the analysis for the MGAs and MEAs and then for the JNDs computed as the standard deviation of the MGAs/MEAs.

#### MGAs & MEAs

The blue data points in Fig. 4 show the MGAs (Fig. 4a) and MEAs (Fig. 4b) for Experiment B averaged over participants. Table 1 shows the slopes averaged over participants. There was no significant difference between the slopes for grasping and manual estimation (t(23) = $-$1.024, p = .316, d = $-$0.209, BF_10_ = 0.344). The Bayes factor provides anecdotal to moderate support that there was no difference between the slopes for the two tasks. A one-sided one-sample *t*-test showed that both the slope for grasping (t(23) = 13.281, p < .001, d = 2.711, BF_+0_ = 5.6$\times$10^+9^) and the slope for manual estimation (t(23) = 12.949, p < .001, d = 2.643, BF_+0_ = 3.4$\times$10^+9^) were significantly larger than zero. The two-sided Bayesian *t*-test showed anecdotal to moderate support for the null hypothesis that the slopes were one (grasping: BF_01_ = 2.838; manual estimation: BF_01_ = 4.322). The equivalent two-sided frequentist *t*-tests were not significant (grasping: t(23) = $-$1.052, p = .304, d = $-$0.215; estimation: t(23) = 0.405, p = .689, d = 0.083).

#### JNDs

The blue data points in Fig. 5 show averaged JNDs for Experiment B computed as the standard deviation of the individual participants’ MGAs (Fig. 5a) and MEAs (Fig. 5b). Table 1 shows the slopes for the JNDs averaged over participants. There was a significant difference between the slopes for grasping and manual estimation (t(23) = $-$3.659, p = .001, d = $-$0.747, BF_10_ = 27.765). The one-sided *t*-test testing if the slopes for grasping were larger than zero was not significant (t(23) = $-$0.263, p = .602, d = $-$0.054, BF_+0_ = 0.178). The Bayes factor indicates that there is little support for the hypothesis that the slope is larger than zero. For manual estimation, the slope was significantly larger than zero (t(23) = 4.425, p < .001, d = 0.903, BF_+0_ = 297.566).

JNDs for manual estimation linearly increased with object height. Together with linearly increasing MEAs this suggests that, in line with previous findings, Weber’s law holds for manual estimation for object sizes between 5 and 20 mm. This does not hold true for grasping. Consistent with previous findings, MGAs increased linearly with object height but JNDs did not but rather remained constant. The linear increase of MGAs with a slope close to one indicates that the constant JNDs cannot be attributed to a lack of resolution. This is also consistent with the more regular pattern of JNDs in Experiment B compared to the noisy pattern in Experiment A.

## Discussion

In two experiments, we investigated grasping of objects with heights ranging from 0.5 to 5 mm (Experiment A) and for heights ranging from 5 to 20 mm (Experiment B). In Experiment A, we found that for very small objects MGAs did not consistently scale with object height, and, consequently, JNDs also did not increase with object height. For manual estimation, however, MEAs increased linearly with object height and so did JNDs thereby reflecting adherence to Weber’s law. As expected, the pattern for MEAs remained the same for the larger objects in Experiment B. Interestingly, while MGAs for the larger objects scaled with object height, contrary to previous findings (Bruno et al., 2016; Uccelli et al., 2021), the JNDs for these objects remained constant.

How do these findings fit in with the predictions of the three explanatory accounts presented in Fig. 1? The results for manual estimation in both experiments are consistent with the predictions of all three accounts. These findings also confirm that even the smallest object heights used in Experiment A were above the perceptual resolution limits for size perception. For the PAM, as explained above, we would consequently expect MGAs to scale with object height for grasping in Experiment A because of the hypothesised higher resolution of the dorsal system. We did not find consistent scaling of MGAs with object height for heights from 0.5 mm to 5 mm. There is an indication that scaling with object height starts towards the end of this range between 4 and 5 mm. The individual participants’ slopes for MGAs in Experiment A (see Figure S1 in the Supplemental material) vary widely from positive to negative. This is markedly different from the more regular pattern of MGAs in Experiment B where all slopes are larger than zero (see Figure S3 in the Supplemental material). While the PAM correctly predicts the absence of Weber’s law for grasping, it does so because it assumes that visual information in the dorsal path is at least as or even more accurate than visual information in the ventral path and not affected by psychophysical principles such as Weber’s law. The notion of high accuracy, however, is inconsistent with the absence of scaling of the MGA while perceptual estimates were sensitive to object height. In this case, the absence of scaling of JNDs with object height does not reflect immunity to Weber’s law but is a consequence of the absence of scaling for MGAs.

In contrast, the digits-in-space hypothesis can explain the lack of scaling of MGAs by assuming that the resolution for positions is lower than the resolution for size (Smeets et al., 2020). Since positions for any object size are not affected by Weber’s law, the digits-in-space hypothesis also correctly predicts the constant JNDs.

Regarding the biomechanical account, the findings are not consistent with predictions derived from an extrapolation of the findings by Bruno et al. (2016) and Uccelli et al. (2021) that Weber’s law can reliably be observed for object sizes below 20 mm. In the absence of scaling of MGAs with object size, Weber’s law can no longer be expected to apply. On the other hand, the findings are consistent with the idea that there are also hard and soft biomechanical constraints for very small objects. These may cause the MGA not to scale with very small object sizes to avoid the effort of producing apertures well below the natural opening between thumb and index finger (i.e., if the muscles are relaxed). If assuming and maintaining a scaled aperture becomes too effortful, a less effortful but still safe strategy might be chosen. Again, if MGAs do not scale with object height, we cannot expect the JNDs to systematically increase with object height.

For Experiment B, the findings by Bruno et al. (2016) and Uccelli et al. (2021) suggest that object sizes in this range can be discriminated. Therefore, the PAM predicts that MGAs scale accurately with object height while JNDs remain constant. This prediction is consistent with our findings. For the digits-in-space hypothesis, the predictions depend on whether position differences for object sizes in this range can be discriminated. If that is the case, then the digits-in-space hypothesis makes—for different reasons—the same predictions as the PAM, i.e., MGAs scale accurately with object height while JNDs remain constant. Otherwise, MGAs should also remain constant. Our finding of increasing MGAs with object height are consistent with the assumption that position data in this range can be discriminated. Therefore, the results for Experiment B are also in line with the predictions by the digits-in-space hypothesis. However, our finding of constant JNDs in Experiment B are not consistent with the predictions of the biomechanical account which is supported by the findings of Bruno et al. (2016) and Uccelli et al. (2021) for objects in this size range. Objects in this range should be well below the upper biomechanical limits and, therefore, the effect of biomechanical constraints should not be strong enough to overshadow the effects of Weber’s law. Yet, we did not observe Weber’s law in the size range between 5 mm and 20 mm. In summary, when looking at the results from Experiments A and B together, our findings align best with the predictions of the digits-in-space hypothesis, and among the three explanatory accounts considered, the digits-in-space hypothesis provides the most straightforward explanation for the observed outcomes.

One interesting question to address is why in Experiment B we could not replicate the findings of the two studies that previously had investigated grasping for objects in the same size range (Bruno et al., 2016; Uccelli et al., 2021). First of all, contradictory findings with regard to Weber’s law in grasping are not uncommon. One notable example are the different findings for bimanual grasping (i.e., grasping an object with both hands) by Ganel et al. (2017) and Hesse, Harrison et al. (2021). The rationale for investigating bimanual grasping is, analogous to that of unimanually grasping small objects, the minimisation of upper biomechanical constraints. Whereas Ganel et al. (2017) did not find adherence to Weber’s law for bimanual grasping, Hesse, Harrison et al. (2021) did. One possible explanation for these divergent findings might be differences in the visibility of the contact points. It has been shown that the visibility of the contact points influences grasping trajectories (Bozzacchi et al., 2018; Volcic & Domini, 2014). In a recent qualification of the digits-in-space hypothesis, Smeets et al. (2022) asserted that if the contact points of one or both digits are not visible, grasping might switch to using size information for action programming. The objects used by Hesse, Harrison et al. (2021) for bimanual grasping were large boxes with side lengths from 16 cm to 40 cm placed closely in front of the participants. The contact points on the sides of the boxes were not directly visible to the participants. Therefore, participants might have used size information instead of position information to program the MGA which in the absence of biomechanical constraints explains why JNDs followed Weber’s law. In contrast, Ganel et al. (2017) used long and thin cylindrical objects (styrofoam rods) with small contact areas on the sides. The participants looked down on these objects that were placed on a table in front of them and moved their hands from a start position on the table to the contact points on the objects. This task and type of stimuli might have encouraged the use of position information which is not affected by Weber’s law. This illustrates that even small differences in experimental tasks and setups might affect the type of information that is used as cue for grasping by the visuomotor system.

A similar reasoning might apply when comparing the findings of the current experiment with the findings of Bruno et al. (2016) and Uccelli et al. (2021). Particularly, our task required participants to grasp objects by placing their digits on the largest surfaces of the objects which were clearly visible. In contrast, in Bruno et al. (2016) and Uccelli et al. (2021) participants grasped disks (1 cm high) placed on a table. Hence, the contact point of the index finger might have been occluded encouraging the use of size information.

If Smeets et al. (2022) correctly assume that when position information is inaccessible or unreliable, the visuomotor system can easily switch to the use of size information, then one could wonder why in the case of Experiment A, where the object sizes might have been below the resolution limit for positions, such a switch did not happen. In that case, we should have seen both scaling of MGAs and JNDs with size. However, as mentioned above, the biomechanical constraints postulated by the biomechanical account could apply independent of whether size or position is the relevant cue for grasping. Experiment A alone does not allow to differentiate between the digits-in-space hypothesis and the biomechanical account. We cannot tell whether the flat MGAs in Experiment A were due to the resolution limits for position data or whether position or size information was available, but scaling was prevented by biomechanical constraints. The results of Experiment B, however, are inconsistent with the predictions of the biomechanical account. The difficulty for the PAM is to explain the absence of scaling for MGAs in Experiment A. If an explanation based on the PAM considered introducing biomechanical constraints, then the lack of scaling of JNDs with object size for grasping would not anymore necessarily indicate a dissociation between perception and action and, thus, could no longer be used as evidence in support of the PAM.

The predictions of the digits-in-space hypothesis were consistent with the results of both Experiment A and B while those of the PAM and the biomechanical account were consistent with only Experiment B or Experiment A, respectively. However, to explain other existing grasping data, the digits-in-space hypothesis would have to allow for a switch from position to size information in certain situations. Our experiments do not allow us to evaluate this assumption. If we look at the data from the various studies that investigated unimanual and bimanual grasping across a wide range of object sizes, a very varied picture emerges. Based on our and previous studies on Weber’s law in grasping, it seems that none of the three accounts can explain all the data without introducing additional assumptions. We can speculate that the default cue for grasping is position data, but in instances where the visibility of contact points is compromised, a switch to the size cue is necessary to explain all data.

More importantly, this study highlights, again, that we cannot regard the MGA as a pure and reliable estimate of object properties (Jeannerod, 1981, 1984) as it is also strongly influenced by task- and observer-related factors, e.g., visibility of contact points and biomechanical constraints (Hesse, Bonnesen et al., 2021; Schenk et al., 2017; Smeets & Brenner, 1999).

## Conclusion

In two experiments, we measured MGAs and JNDs for grasping of objects with heights ranging from 0.5 to 20 mm, and compared the results to the predictions of three explanatory accounts of grasping. We found that the predictions of the digits-in-space hypothesis best aligned with the results of both experiments suggesting that the relevant cue for programming of MGAs is position rather than size. This together with the role of modulating factors, such as the visibility of contact points, biomechanical constraints and task demands, provides consistent explanations for the outcomes of studies investigating Weber’s law in a wide range of uni- and bimanual grasping tasks without the need to assume different pathways for the processing of visual information in perception and action.

### Supplementary Information

Below is the link to the electronic supplementary material.

Supplementary file 1 (pdf 12473 KB)

## Data Availability

Supplemental data is provided as supplemental material: SupplementalMaterial.pdf. The data presented here are available online from the Open Science Framework (OSF) with this link: https://osf.io/jv7as/.
