# Supplementary material for: Grasping tiny objects
Source: Psychol Res. 2024 Mar 30;88(5):1678–90. doi: 10.1007/s00426-024-01947-8 (PMC11281983; doi:10.1007/s00426-024-01947-8)
Supplement: Supplementary file 1 — (pdf 12473 KB) [file 426_2024_1947_MOESM1_ESM.pdf]

Supplemental material for "Grasping tiny objects"

Martin Giesel[1*], Federico De Filippi[1,2] and Constanze Hesse[1]

[1*]School of Psychology, University of Aberdeen, William Guild Building, Aberdeen, AB24 3FX, UK.
[2]School of Psychology and Neuroscience, University of St Andrews, St Mary's Quad, South Street, St Andrews, KY16 9JP, UK.

*Corresponding author(s). E-mail(s): martin@martingiesel.net;
Contributing authors: fdf1@st-andrews.ac.uk; c.hesse@abdn.ac.uk;

Contents

1 Experiment A **2**

1.1 MGAs 2

1.2 MEAs 3

2 Experiment B **4**

2.1 MGAs 4

2.2 MEAs 4

1 Experiment A

1.1 MGAs

Figure S1 shows the maximum grip apertures (MGAs, grey circles) for each participant and object height to which regression lines were fitted (red and cyan lines). See main text for details.

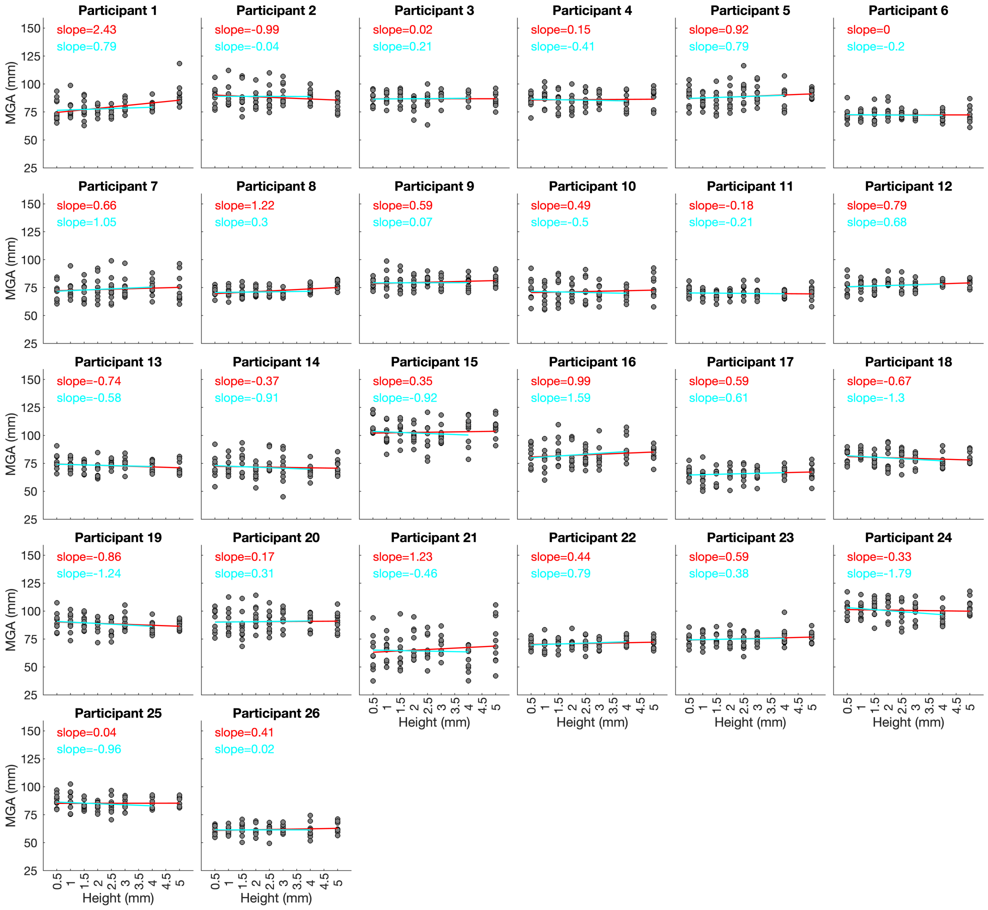

Fig. S1 MGAs for all participants and object heights (grey circles). The red lines are regression lines fitted to each participants MGAs. Insets in red indicate the slope of the red line. The cyan line is the regression line fitted to object heights from 0.5–4 mm, i.e., excluding the highest object (5 mm). The cyan inset indicates the slope of the cyan regression line.

1.2 MEAs

Figure S2 shows the maximum estimation apertures (MEAs, grey circles) for each participant and object height to which regression lines were fitted (red and cyan lines). See main text for details.

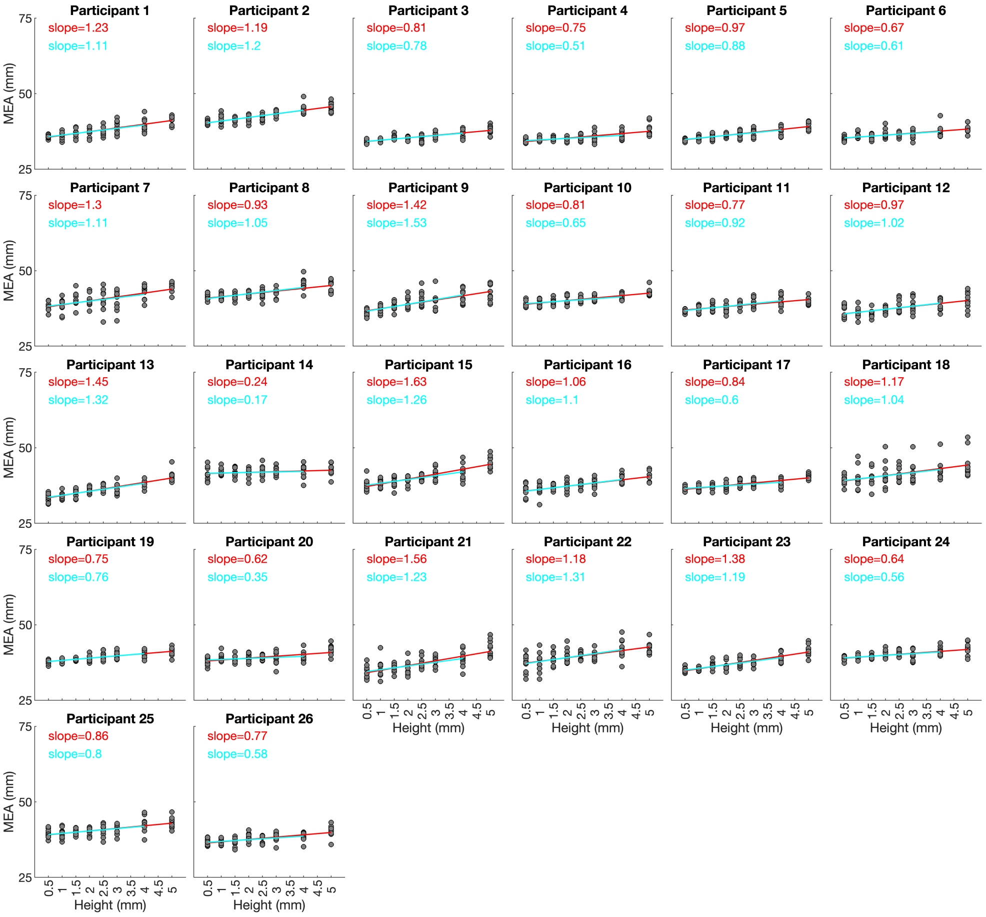

Fig. S2 MEAs for all participants and object heights (grey circles). The red lines are regression lines fitted to each participants' MEAs. Insets in red indicate the slope of the red line. The cyan line is the regression line fitted to object heights from 0.5–4 mm, i.e., excluding the highest object (5 mm). The cyan inset indicates the slope of the cyan regression line.

2 Experiment B

2.1 MGAs

Figure S3 shows the MGAs (grey circles) for each participant and object height to which a regression line was fitted (red line).

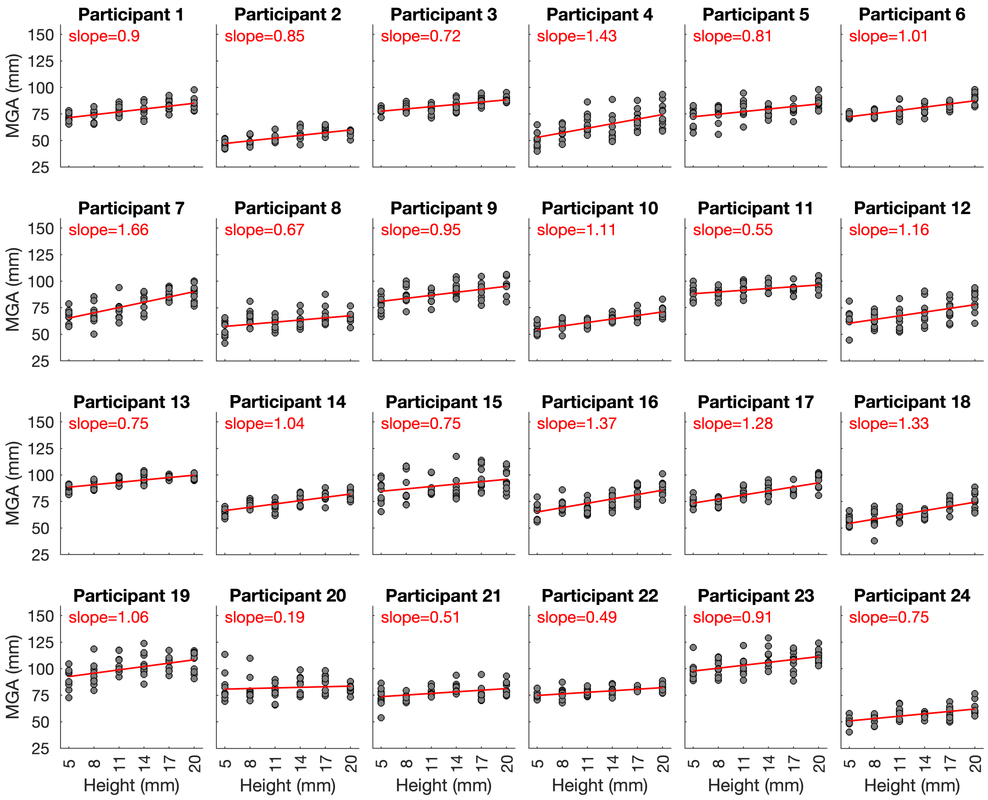

Fig. S3 MGAs for all participants and object heights (grey circles). The red lines are regression lines fitted to each participants' MGAs. Insets in red indicate the slope of the red line.

2.2 MEAs

Figure S4 shows the MEAs (grey circles) for each participant and object height to which a regression line was fitted (red line).

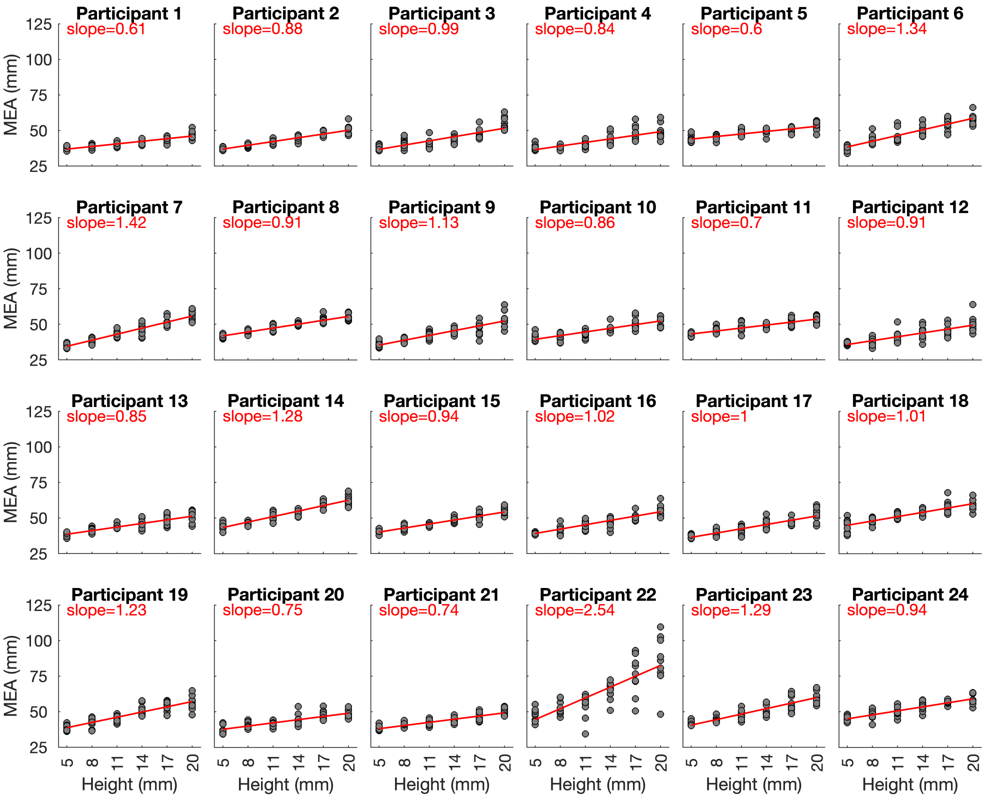

Fig. S4 MEAs for all participants and object heights (grey circles). The red lines are regression lines fitted to each participants' MEAs. Insets in red indicate the slope of the red line.
